# Low Temperature Processed Complementary Metal Oxide Semiconductor (CMOS) Device by Oxidation Effect from Capping Layer

**DOI:** 10.1038/srep09617

**Published:** 2015-04-20

**Authors:** Zhenwei Wang, Hala A. Al-Jawhari, Pradipta K. Nayak, J. A. Caraveo-Frescas, Nini Wei, M. N. Hedhili, H. N. Alshareef

**Affiliations:** 1 Materials Science and Engineering, King Abdullah University of Science & Technology (KAUST), Thuwal 23955-6900, Saudi Arabia; 2 Department of Physics, King Abdulaziz University, Jeddah 21589, Saudi Arabia

## Abstract

In this report, both *p-* and *n-*type tin oxide thin-film transistors (TFTs) were simultaneously achieved using single-step deposition of the tin oxide channel layer. The tuning of charge carrier polarity in the tin oxide channel is achieved by selectively depositing a copper oxide capping layer on top of tin oxide, which serves as an oxygen source, providing additional oxygen to form an *n*-type tin dioxide phase. The oxidation process can be realized by annealing at temperature as low as 190°C in air, which is significantly lower than the temperature generally required to form tin dioxide. Based on this approach, CMOS inverters based entirely on tin oxide TFTs were fabricated. Our method provides a solution to lower the process temperature for tin dioxide phase, which facilitates the application of this transparent oxide semiconductor in emerging electronic devices field.

Complementary metal oxide semiconductor (CMOS) architecture is the most fundamental building block in many integrated circuits (ICs) largely due to its low power consumption and efficient noise control^1^,^2^. In the field of transparent electronics, there is a need to develop CMOS devices using transparent oxide thin film transistors (TFTs)^3^,^4^. In the last decade, both *n-* and *p-*type transparent semiconducting oxides (TSOs) have been studied extensively^5^. Transparent *n-*type oxide based TFTs using tin dioxide (SnO_2_)^6^, indium oxide (In_2_O_3_)^7^, zinc oxide (ZnO)^8^ and amorphous indium-gallium-zinc-oxides (IGZO)^9^ as active layers have been demonstrated with good electrical properties. Similarly, *p-*type oxide TFTs, based on tin monoxide (SnO)^10^,^11^ and copper-based oxides (CuO and Cu_2_O)^12^,^13^,^14^,^15^ have been recently reported with encouraging *p-*type performance^10^,^14^,^16^,^17^,^18^. In spite of these progressive developments in oxide TFT field, however, the application of TFT-based CMOS in ICs is limited by the incompatible processing procedures required by the *n-* and *p*-type oxide TFTs^19^. Depending on the valence state of tin, semiconducting oxides of tin have been shown to be either *n-*type (for SnO_2_) or *p-*type (for SnO). Each of them exhibits decent electrical performance, and possesses a large optical band gap and, accordingly, good transparency in the visible range. Yet, different deposition parameters and post-deposition annealing (PDA) conditions lead to a complicated fabrication process to integrate these two oxides in the same device.

For both oxides of tin, the PDA process is always required in order to crystallize the amorphous as-deposited films. For *p*-type SnO phase, precisely controlling of the PDA process and maintaining it at moderate condition is of significant importance, since SnO is a thermodynamically metastable phase^20^,^21^. In particular, although PDA helps to crystallize the as-deposited amorphous SnO, it can also transform SnO to *n-*type SnO_2_ phase if over oxidized for long duration or at a higher temperature. In contrast, for *n*-type SnO_2_ phase, it is reported that PDA temperature above 300°C is required, which limits the application of SnO_2_ on flexible substrates^22^. Thus, different PDA processes required by SnO and SnO_2_ phase formation inhibit the integration of these two promising TSOs into the same device.

Recently, we reported that simultaneous growth of *n-*type SnO_2_ and *p-*type SnO films could be achieved by using solution-derived and atomic layer deposited (ALD) aluminum oxide (Al_2_O_3_) as the dielectric layers^19^. It was shown that the presence of large number of hydroxyl groups in the solution-derived Al_2_O_3_ can be considered as an additional oxygen source, which contributed to the formation of SnO_2_ phase at low annealing temperature of 210°C. Here, we report a novel approach of transforming SnO to SnO_2_ phase at PDA temperature as low as 190°C, which facilitates the fabrication of both *p-* and *n-*type TFTs on the same dielectric layer. Such low processing temperature was achieved by using a dual active layer structure (capping layer/SnO layer). We found previously^21^ that using Cu_2_O as a capping layer on top of SnO could regulate oxidation of the exposed surface of the SnO *p*-channel, on one hand, and control oxygen diffusion into the underlying SnO*_x_* film, on the other. The Ellingham diagram for tin oxides and copper oxides is shown in Figure S1. In this type of diagram, oxides located in higher position could oxidize the ones below them. In this system, it is clear that copper oxides can oxidize the underlying tin oxides and would preferentially form SnO_2_ over a large temperature range. In this work, we aim to manipulate the Cu_2_O/SnO bilayer scheme to oxidize the *p*-type SnO layer forming *n-*type SnO_2_ at temperature much lower than normally required by annealing SnO in air. The performance of *n-*type bilayer TFT was optimized by controlling the thicknesses of both SnO layer and the capping layer. Finally, transparent CMOS inverters were fabricated by combining our *p-* and *n-*type TFTs. Our method provides an alternative solution to lower the process temperature of high-temperature SnO_2_ phase, which can enlarge the selection range of TSOs applied on the temperature sensitive flexible substrates.

## Results

### Devices performance

**Figure 1:**
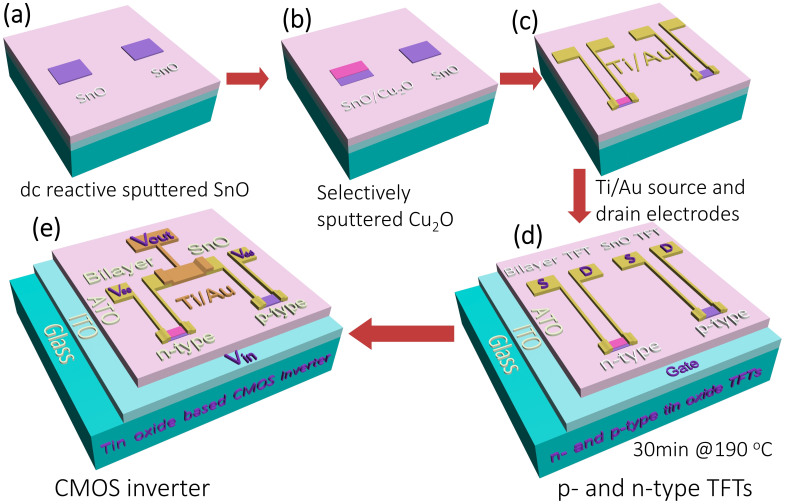
Flow diagram for TFTs and CMOS inverters fabrication. The detailed structures of TFTs and CMOS inverters are shown in (d) and (e).

**Figure 2:**
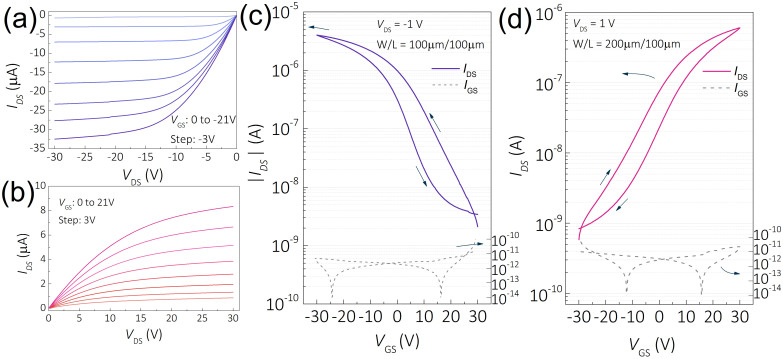
Output and transfer characteristics of *p-* and *n-*type TFTs. Output characteristics for (a) *p-*type SnO TFT and (b) *n-*type bilayer TFT; Transfer characteristics for (c) *p-*type SnO TFT and (d) *n-*type bilayer TFT.

Fabrication process flow for both *n-* and *p-*type TFTs and tin oxide TFTs based CMOS inverter is shown in Figure 1. The capacitance and current-voltage curve for the ATO dielectric is presented in Figure S2, the average capacitance is found about 55 nFcm^−2^. For the *p-*type TFT, single SnO layer was used as active layer, while Cu_2_O/SnO bilayer was used for the *n-*type TFT, as depicted in Figure 1(d) and (e). The output and transfer characteristic curves of both TFTs are presented in Figure 2(a)–(d). The output characteristics of the single layer SnO TFT is shown in Figure 2(a), which exhibits *p*-type conductivity since the source to drain current (*I*_DS_) generated under a negative gate voltage (*V*_GS_). The output characteristics of TFT with Cu_2_O/SnO bilayer channel is presented in Figure 2(b). In contrast to the single layer SnO TFT, the TFT with Cu_2_O/SnO bilayer shows an *n-*type conductivity, since the *I*_DS_ generated under a positive *V*_GS_. In both cases, distinct linear and saturation regions can be identified. Current crowding was not observed in the linear region of either output characteristics, indicating Ohmic contact between titanium/gold electrodes and SnO or bilayer channel.

The transfer characteristics of *p-* and *n*-type TFTs are presented in Figure 2(c) and (d), which were measured in the linear operation region under a fixed *V*_DS_ of −1 V and +1 V, respectively. With reference to the TFT characterization procedure recommended by J. F. Wager^23^, linear-region field-effect mobility (*μ*_FE_) were calculated from the transfer characteristics measured with various W/L ratios ranging from 0.05 to 10. Multiple devices were evaluated for each *W*/*L* ratio, the results are shown in Figure S3. The field-effect mobility, *μ*_FE_, for *p-* and *n-*type TFTs are calculated to be 2.39 ± 0.13 and 0.23 ± 0.03 cm^2^ V^−1^ s^−1^, respectively. Multiple cycles of dual-sweep scans were performed on each type of TFTs and the results are shown in Figure S3. Both types of TFTs exhibited retracable transfer curves under forward or backward sweep of gate voltage which indicates that both types of TFTs belong to type II: non-equilibrium, steady-state behavior, as suggested by J. F. Wager^23^. The performance of bilayer TFTs with various SnO layer thicknesses and Cu_2_O/SnO thickness ratios are presented in Figure S4. These results show that the device performance strongly depends on the thickness of Cu_2_O and SnO layers. The optimized bilayer TFT was chosen to build CMOS inverters.

The gate leakage current of both *n-* and *p-*channel devices are very low (~10^−11^ A, Figure 2), which indicates that the effect of gate leakage current on mobility estimation can be ignored. For *p-*channel SnO TFT, the threshold voltage (*V*_th_), sub-threshold swing (SS) and on-current to off-current ratio (*I*_on_/*I*_off_) are 0.87 V, 7.5 V/dec and ~10^3^, respectively. For n-channel device, *V*_th_, SS and *I*_on_/*I*_off_ were estimated to be 0.58 V, 12 V/dec and ~10^3^, respectively. We believe the TFTs performance can be further optimized by changing gate dielectric, tuning the oxygen partial pressure (Opp) when depositing the copper oxide, or by further optimizing the annealing temperature.

**Figure 3:**
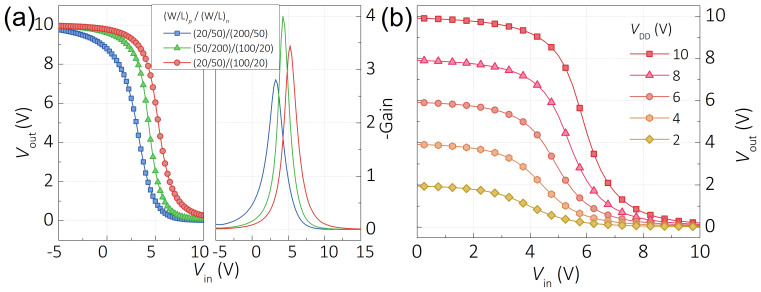
Performances of CMOS inverters. (a) Voltage transfer and gain characteristics of CMOS inverters with variable channel size ratios. (b) Voltage transfer curves of optimized CMOS inverter.

The schematic illustration of our CMOS inverter is presented in Figure 1(e). The ITO bottom gate layer was connected to the input terminal as *V*_in_. The source electrode of *p-*type TFT was used as the *V*_dd_ terminal. The source electrode of *n*-type TFT was applied as the *V*_ss_ terminal, which was connected to an Agilent high performance ground unit. Finally, the output terminal (*V*_out_) was built by connecting the drain electrodes of both *p-* and *n-*type TFTs. In order to adjust the transition voltage (*V*_M_, maximum gain voltage) of CMOS inverter, load ratio (*β* = *μ* × *W*/*L*) of each type TFT was accurately measured and calculated. Finally, by selecting large (*W*/*L*)_n_/(*W*/*L*)_p_ ratio, compatible *p-* and *n-*type load ratios (*β*_n_/*β*_p_ ~ 1) were achieved and the *V*_M_ was located at about *V*_dd_/2. The voltage transfer curves (VTC) of the selected CMOS inverters are shown in Figure 3(a). The *V*_M_ is sensitive to the *β_n_/β_p_* and the *V*_th_ of each type oxide TFT. Gain of CMOS inverter was calculated by evaluating the negative slope (−d*V*_out_/d*V*_in_) of each VTC curve and the results are shown in Figure 3(a), our CMOS inverters show a maximum gain of ~4. The VTC curve of the optimized CMOS inverter under linearly increased *V*_dd_ is presented in Figure 3(b). We attribute the low gain value to the large SS and the low *I_on_/I_off_* of TFTs, which may be optimized by replacing the dielectric layer, stacking a passivation layer on top of device.

### Materials Characterization

**Figure 4:**
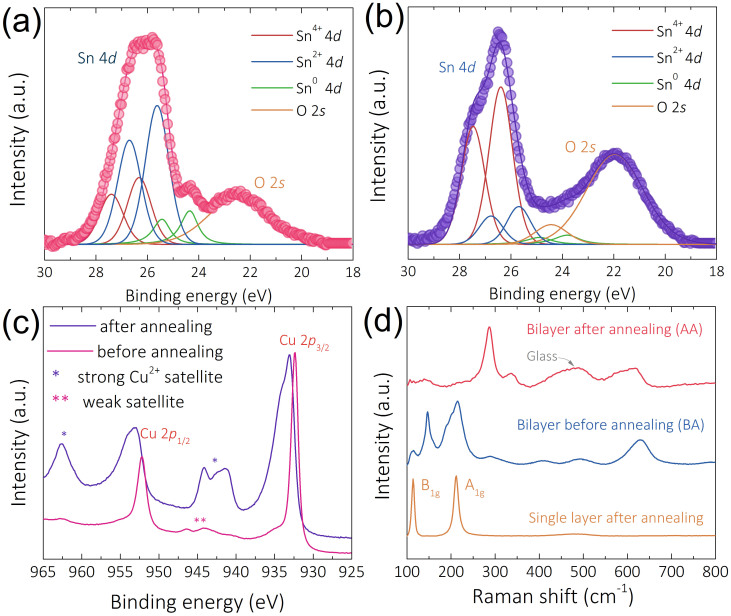
Materials characterizations for TFT channel layers. XPS Sn 4*d* peaks of (a) before and (b) after annealing bilayer sample; (c) XPS Cu 2*p* peaks of bilayer sample. (d) Raman spectra of before and after annealing bilayer samples and SnO single layer sample.

Recently, we have demonstrated a detailed investigation of the origin of *p-*type transport behavior in SnO channel TFTs^10^. Here we focus on the origin of *n*-type transport behavior in Cu_2_O/SnO bilayer TFTs. To investigate the oxidation state of tin in Cu_2_O/SnO bilayer, X-ray photoelectron spectroscopy (XPS) was performed on the before annealing (BA) and after annealing (AA) bilayer samples, which were prepared under the same conditions used in the TFTs fabrication (i.e., annealing was performed at 190°C for 30 min in air). The XPS spectra of the Sn 4*d* peaks of BA and AA samples are presented in Figure 4(a) and (b), respectively. It is reported that the Sn 4*d* peak is a doublet and consists of Sn 4*d*_3/2_ and Sn 4*d*_5/2_ located at binding energy of 27.3 and 26.2 eV, respectively^24^,^25^. The deconvolution of both Sn 4*d* peaks of BA and AA samples show Sn 4*d*_3/2_ and Sn 4*d*_5/2_ doublet peaks, with a chemical shift of ~0.7 eV between doublet peaks, which is consistent with the report from Themlin *et al*.^26^ For Sn^4+^, Sn 4*d*_3/2_ and Sn 4*d*_5/2_ peaks are located at 27.4 and 26.3 eV, respectively; in contrast, for Sn^2+^, Sn 4*d*_3/2_ and Sn 4*d*_5/2_ peaks are located at 26.7 and 25.6 eV, respectively. This is a clear evidence of the co-existance of both SnO and SnO_2_ phases in our bilayers. In case of both BA and AA samples, Sn 4*d* peak corresponding to metallic tin could be observed, this is nature of direct current (dc) reactive magnetron sputtering thin film and is consistent with our previous report^10^. Interestingly, the atomic content (in at%) of Sn^4+^, Sn^2+^ and Sn^0^ are determined to be 28, 62 and 10%, respectively in BA sample, and 78, 16 and 6%, respectively in AA sample. In the BA sample, the dominant phase is determined to be SnO. However, the content of *n-*type SnO_2_ phase significantly increases in AA sample, and becomes dominant (~78 at%) after the PDA process, which we believe is the origin of *n-*type transport behavior in the bilayer TFTs. The analysis result of XPS Sn 3*d* spectra of BA and AA sample is in agreement of XPS Sn 4*d* spectra (Figure S5).

XPS Cu 2*p* peaks were obtained to investigate the phase tranformation of copper in bilayer sample during the PDA process. Both XPS Cu 2*p* doublets of BA and AA samples are shown in Figure 4(c). It is reported that XPS Cu 2*p* doublet consists of two main peaks at 952 (Cu 2*p*_1/2_) and 932 eV (Cu 2*p*_3/2_) and some satellite peaks may exist depending on the oxidation state of Cu^27^,^28^. According to our XPS results, doublets attributed to Cu 2*p*_1/2_ and Cu 2*p*_3/2_ can be explicitly detected in both samples. For the BA sample, peaks located at 932.2 and 952.1 eV are attributed to the core level Cu 2*p*_3/2_ and Cu 2*p*_1/2_, respectively. In addition to these two peaks, some weak satellite peaks can also be observed at ~945 and ~962 eV. Similar results have also been reported by Barreca *et al.*^27^, indicating that copper oxide in BA sample exists as Cu_2_O phase. For Cu 2*p* spectra of AA sample, doublet peaks with ~1 eV chemical shift from the BA one was observed. Main peaks located at 933.2 and 953.1 eV, these two peaks correspond to Cu 2*p*_3/2_ and Cu 2*p*_1/2_ of the CuO phase, respectively^29^. In addition to these two main peaks, three other intense satellite peaks located at 941.2, 944.2 and 962.6 eV were observed in the AA sample. These peaks were reported as shake-up satellites^30^,^31^, a phenomenon where the emitted photoelectrons encounter the valence electrons that are being excited to higher energy level(s). This process would decrease the kinetic energy of these photoelectrons, thus satellite peaks with higher binding energy will appear in XPS spectra, correspondingly. The appearance of these intense satellite peaks is attributed to the completely oxidized CuO phase. Therefore, after the PDA process, higher oxidized state CuO is formed.

Raman spectra of BA and AA samples are shown in Figure 4(d). In case of the BA sample, peaks at 112, 147, 213 and 631 cm^−1^ corresponding to the Cu_2_O phase were observed, similar results were also reported by Zoolfakar and Solache-Carranco *et al.*^28^,^32^ For the AA sample, Raman peaks located at 287, 340 and 621 cm^−1^ were observed, which were assigned to the CuO phase, similar results were reported by Zoolfakar and Rashad *et al.*^28^,^33^ The phase analysis from Raman spectrum for BA and AA sample is consistent with the XPS results. Raman spectra for post-annealed single layer sample is also shown in Figure 4(d), in which, two peaks at 112.1 and 211 cm^−1^ can be observed, which were assigned to the A_1g_ and B_1g_ vibration modes of tin monoxide, respectively^34^.

UV-Vis transmission spectra of SnO single layer and Cu_2_O/SnO bilayer are presented in supplementary information (Figure S6). Films were deposited on glass substrates under the same condition as the actual device. The transmittances of single layer and bilayer samples are above 85% and 54% respectively, in wavelength range from 400 to 700 nm.

**Figure 5:**
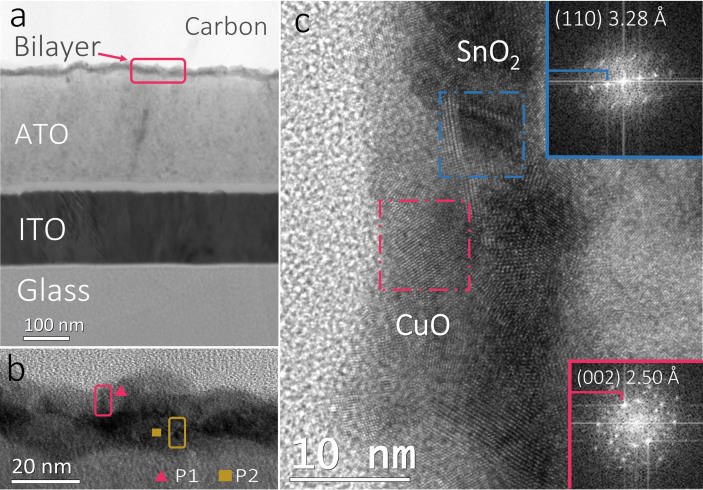
Cross-sectional TEM characterizations for bilayer sample. TEM micrograph of (a) the stack structure of glass-substrate/ITO (150 nm)/ATO (220 nm)/bilayer and (b) details of copper oxide (pink rectangular) and tin oxide (yellow rectangular) zones, EDS point analysis positions were labeled as P1 and P2. (c) High resolution TEM micrograph of bilayer sample, FFT analysis of selected zones are depicted as the insets.

High resolution cross-sectional transmission electron microscopy (TEM) was performed to investigate the structure and phase composition of the post annealed bilayer sample, the results are shown in Figure 5. The cross-sectional TEM micrograph of the bilayer device is presented in Figure 5(a), from which the stack structure of glass-substrate/ITO (150 nm)/ATO (220 nm)/bilayer can be seen clearly. High resolution TEM cross-sectional micrograph is shown in Figure 5(c), in which copper oxide and tin oxide layer stack can be seen as a well-defined bilayer structure. Fast Fourier transform (FFT) analysis was performed in selected areas marked in Figure 5(c) and the results are shown as the insets. The FFT analysis for tin oxide zone shows inter-planar spacing (*d*) value of ~3.28 Å which is close to the *d*_110_ of SnO_2_ (JCPDS card No. 00-041-1445). For copper oxide, *d* value of ~2.50 Å could be measured from the FFT pattern, which is close to the *d*_002_ in CuO (JCPDS card No. 00-002-1040). Figure 5(b) shows the larger scale TEM micrograph for the bilayer sample, pink rectangular indicates the CuO layer and the yellow one for SnO_2_ layer. Energy dispersive X-ray spectrometer (EDS) point analysis was performed on the selected small zones labeled as P1 and P2 in the TEM image. The obtained results show only copper peaks in P1 and tin peaks can be detected in P2 (Figure S7). This result is consistent with the FFT analysis, which confirms the bilayer sample in fact exists as CuO/SnO_2_ bilayer stacking structure. High resolution cross-sectional TEM micrograph of as-deposited bilayer sample is shown in Figure S8, in which both the CuO_2_ and SnO layer are found to be amorphous.

## Discussion

It has been reported that the metastable SnO phase could be oxidized to SnO_2_ at temperature higher than 300°C in air^22^. However, for the bilayer case, we found that temperature as low as 190°C is enough to realize this oxidation process. There are three points that can help us understand the low-temperature phase transition from SnO to SnO_2_ in our bilayer films. Firstly, according to the bilayer structure (Figure 5, S7 and S8), copper oxide layer is located directly above tin oxide layer, which makes copper oxide a good candidate as the oxygen source. Secondly, the tin oxide layers were deposited and annealed at the same time, but the as-deposited single layer tin oxide TFT was only transformed to *p*-type from the same annealing, which indicates the *n*-type behavior of bilayer TFT comes from the additional oxidation effect from the copper oxide capping layer. Finally, according to the Ellingham diagram (Figure S1), the corresponding lines of copper oxides are located above the lines for tin oxides within a large temperature range, which confirms the thermodynamical possibility of copper oxide serving as oxygen source and oxidizing the underlying tin oxide layer. Combining the XPS, Raman and TEM results, we can conclude that during the PDA process the surface cuprous oxide (Cu_2_O) was oxidized to CuO and oxygen atoms from copper oxide layer diffused into the underlying tin oxide layer, forming *n*-type tin dioxide phase at temperature as low as 190°C.

In conclusion, both *p-* and *n-*type tin oxide based TFTs were acquired from the same low temperature PDA process in air. *N-*type bilayer TFT was achieved by applying dual-active-layer structure, i.e. Cu_2_O/SnO channel layer, while the SnO single layer TFT shows *p-*type polarity. Based on these tin oxides TFTs, CMOS inverters were successfully fabricated. Our materials characterization results demonstrate that the copper oxide layer served as the oxygen source, which oxidized the underlying as-deposited tin oxide layer, forming *n-*type SnO_2_ phase at temperature as low as 190°C. Therefore, by selectively depositing a capping layer, variable valence states of tin oxides were achieved simultaneously, which enables the low-temperature tuning of transporting polarity of tin oxides. Our method provides an alternative solution to lower the process temperature of SnO_2_, which normally requires an annealing temperature over 300°C^22^. This approach facilitates the application of transparent semiconductors in emerging electronic devices.

## Methods

### Device fabrication

Commercial indium tin oxide (ITO) and a bilayer of aluminum oxide and titanium oxide (ATO) deposited by atomic layer deposition on glass substrates were used as the gate electrode and dielectric, respectively. The glass substrates with ITO and ATO films were purchased from Planar Systems Inc., Finland. Substrates were cleaned sequentially by acetone, isopropanol and de-ionized water using an ultrasonic cleaner (Branson 3510, Switzerland), for 15 min in each solution and finally dried by nitrogen gas. Tin oxide and copper oxide thin films were deposited by direct current reactive magnetron sputtering (Angstrom Engineering Inc, Canada) at room temperature using a mixture of argon and oxygen gases. Tin oxide films were deposited at an oxygen partial pressure (Opp) of 9%, dc power of 20 W and pressure of 1.8 mTorr. After deposition of tin oxide films, half of substrate was covered by Kapton tape as protection mask for selective deposition of the capping layer. Copper oxide films were then deposited at Opp of 10%, dc power of 50 W, and pressure of 4.5 mTorr. 2-inch tin metal target (purity ~99.99%) and 2-inch copper target (purity ~99.99%) were used for sputtering. The growth rates of tin oxide and copper oxide are ~0.65 and 1 Å/s, respectively. Titanium (10 nm) and gold (70 nm) source and drain electrodes were deposited by e-beam evaporation. Both channel and electrode layers were patterned by lift-off process using 1.4 *μ*m photoresist (AZ 1512HS from MicroChemicals). Multiple channel widths and lengths were patterned in each substrate, varying from 10 to 200 *μ*m. Post-deposition annealing of the devices was performed at 190°C for 30 min in a tube furnace (Thermo Scientific) in air. SnO single layer and Cu_2_O/SnO bilayer thin films were prepared under the same deposition condition for materials characterization.

### Device and material characterizations

The capacitance curve for the ATO dielectric was measured by a capacitance meter (Agilent E4981A). The electrical performance of *p-* and *n-*type TFTs and CMOS inverters were characterized at room temperature in dark using a semiconductor device analyzer (Agilent B1500A) and a microprobe station (Summit-11600 AP, Cascade Microtech). The chemical composition of the Cu_2_O/SnO bilayer films was analyzed by x-ray photoelectron spectroscopy (XPS) using an Axis Ultra DLD spectrometer (Kratos Analytical, UK). Raman spectra were analyzed at room temperature at wavenumber range from 100 to 800 cm^−1^ by LabRAM ARAMIS Raman Microscope (Horiba Scientific) and a 473 nm cobalt laser source was used for excitation. Cross-sectional TEM sample was prepared by a focused ion beam (FIB) from Quanta 3D FEG (FEI). About 500 nm amorphous carbon layer was deposited by a carbon coater (Emitech K950X) as protection layer before performing the cross-sectional sample preparation by FIB. High resolution TEM image of bilayer sample was investigated by a Titan ST (FEI) transmission electron microscope. The UV-Vis transmittance spectra was measured by Evolution 600 UV-Vis Spectrophotometer (Thermo Scientific).

## Author Contributions

Z.W., H.A.A. and H.N.A. designed the research, analyzed the data and co-wrote the report. Z.W., P.K.N. and J.A.C. carried out the device fabrications and characterizations. N. W. contributed for the TEM imaging. M.N.H. contributed with film characterizations by XPS.

## Supplementary Material

Supplementary Information Supplementary Information
