# Supplementary material for: Low Temperature Processed Complementary Metal Oxide Semiconductor (CMOS) Device by Oxidation Effect from Capping Layer
Source: Sci Rep. 2015 Apr 20;5:9617. doi: 10.1038/srep09617 (PMC4402970; doi:10.1038/srep09617)
Supplement: Supplementary Information [file srep09617-s1.pdf]

SUPPLEMENTARY INFORMATION

Low Temperature Processed Complementary Metal Oxide Semiconductor (CMOS) Device by Oxidation Effect from Capping Layer

Zhenwei Wang[1], Hala A. Al-Jawhari[2], Pradipta K. Nayak[1], J. A. Caraveo-Frescas[1], Nini Wei[1], M. N. Hedhili[1], and H. N. Alshareef [1,*]

[1]Materials Science and Engineering, King Abdullah University of Science & Technology (KAUST), Thuwal 23955-6900, Saudi Arabia

[2]Department of Physics, King Abdulaziz University, Jeddah 21589, Saudi Arabia

*email: husam.alshareef@kaust.edu.sa

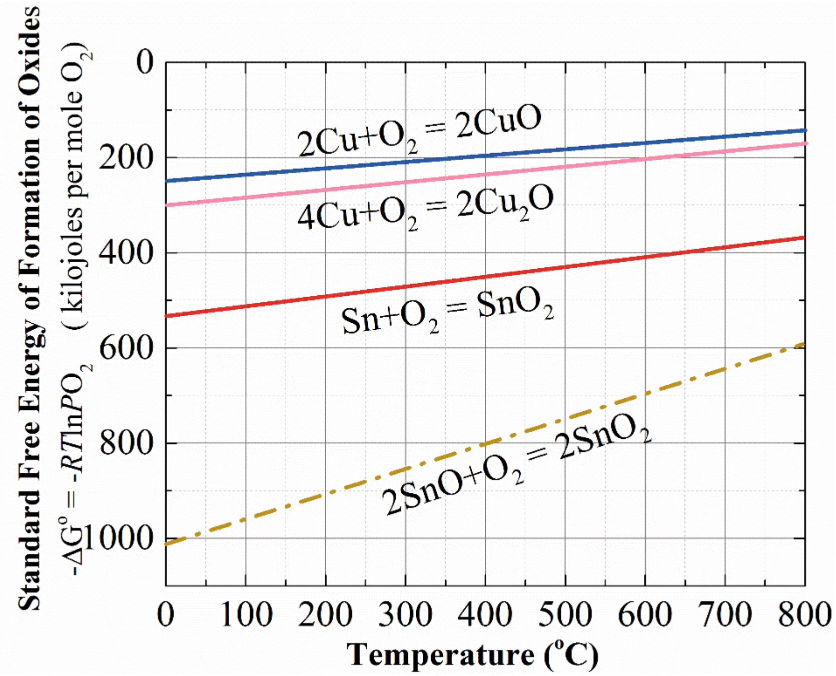

Figure S1. The Ellingham diagram for tin oxides and copper oxides. Thermodynamic data adopted from reference [1].

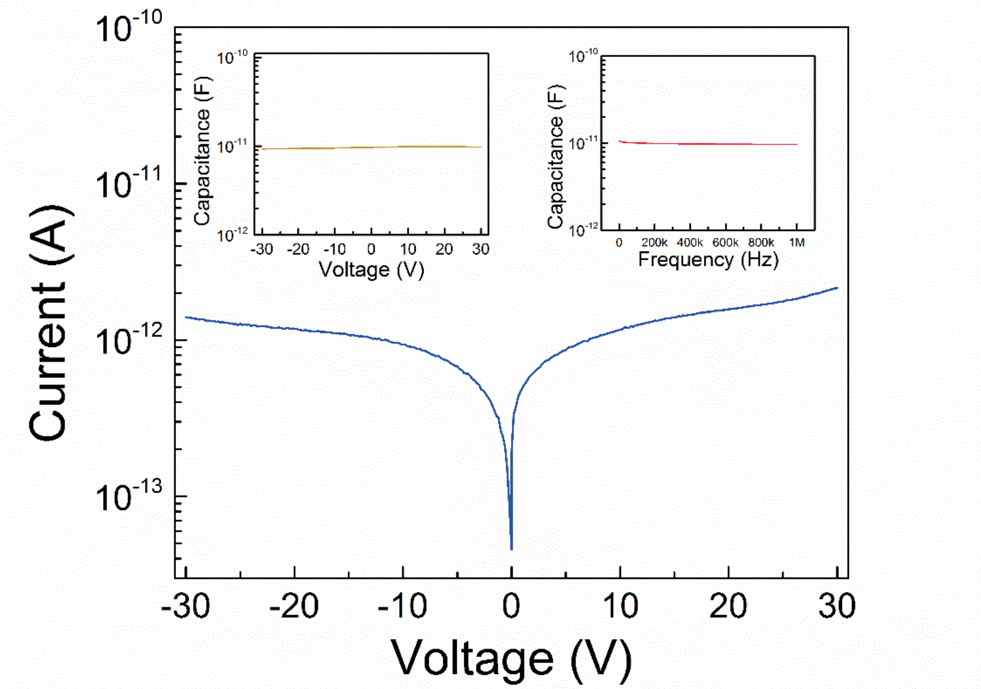

Figure S2. Current-voltage characteristics of the ATO dielectrics. The capacitance-voltage curve is shown in the left inlet, which was measured at 1 MHz and the capacitance-frequency curve is shown in the right inlet, which is measured at a bias of 1 V. The contacting area of metal-insulator-metal is about 25000 μm^2 and the average capacitance is found about 55 nFcm^{-2}.

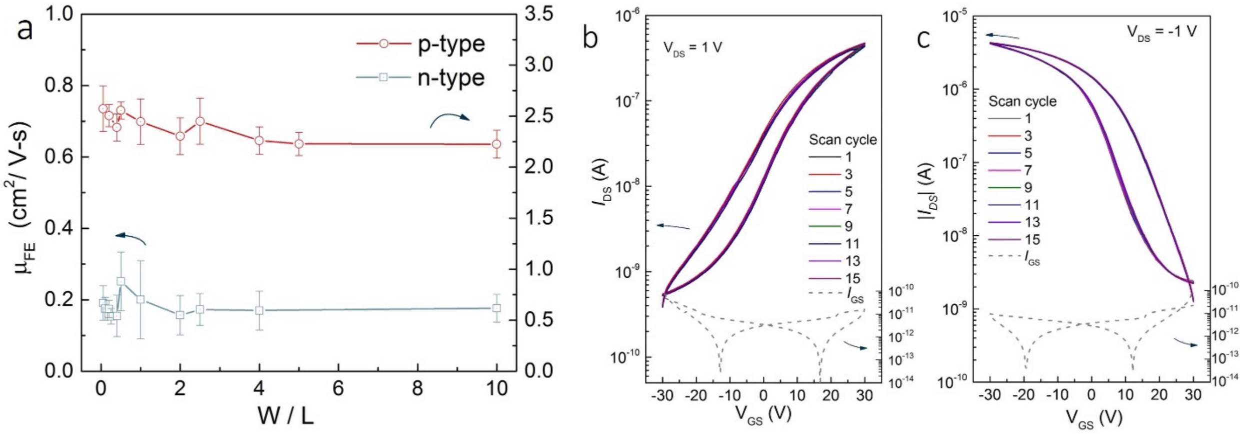

Figure S3. Mobility evaluated from multiple channel *W/L* ratios and reliability test of both *p*- and *n*-type TFTs. Field-effect mobility of both *p*- and *n*-type TFTs with multiple *W/L* ratios range from 0.05 to 10 are shown in (a). Multiple cycles of dual-sweep transfer characteristic curve of (b) *n*- and (c) *p*-type TFTs.

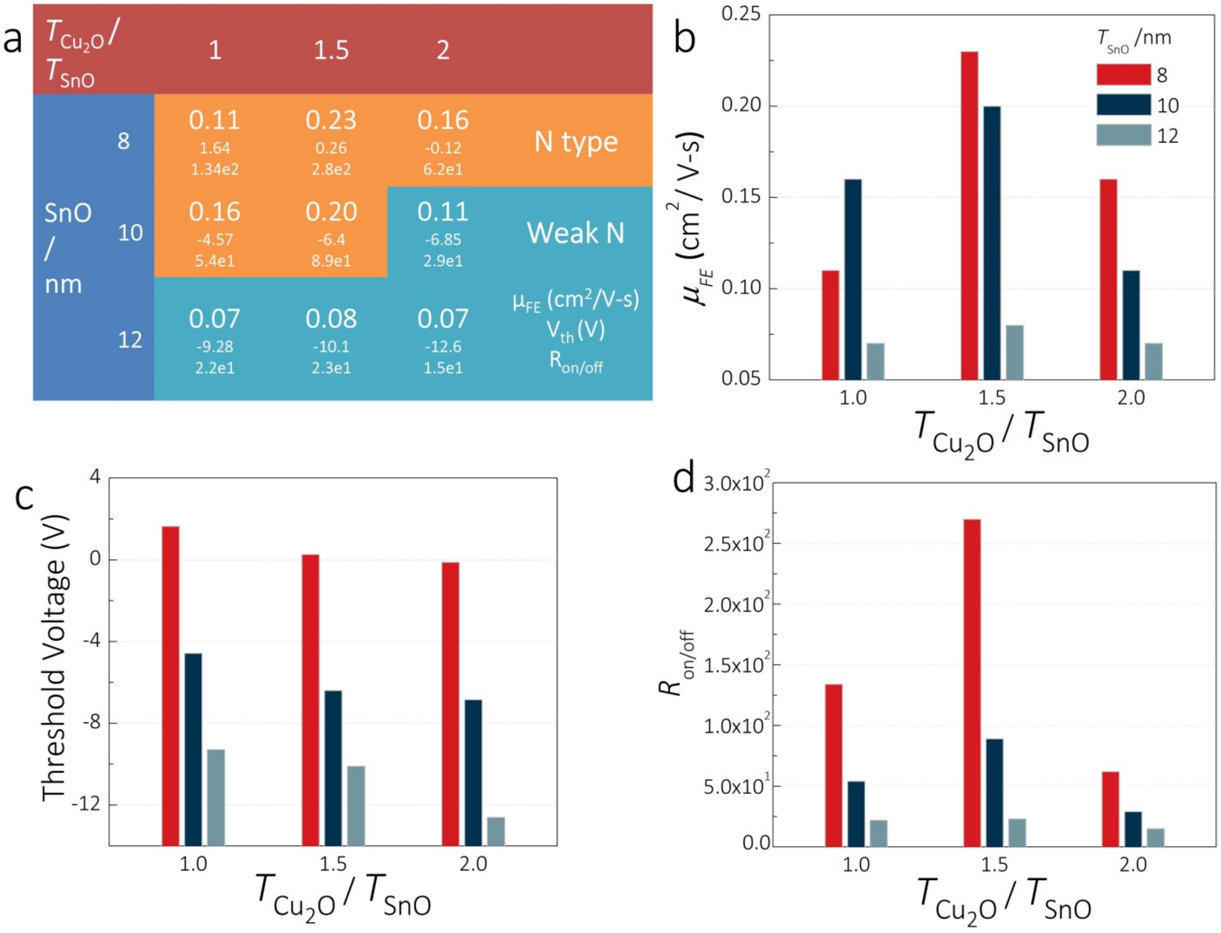

Figure S4. Performance of TFTs with various SnO and Cu_2O thickness combinations. Thickness combinations design of bilayer sample is presented in (a). Specific parameter comparisons of (b) field-effect mobility, (c) threshold voltage and (d) on current to off current ratio.

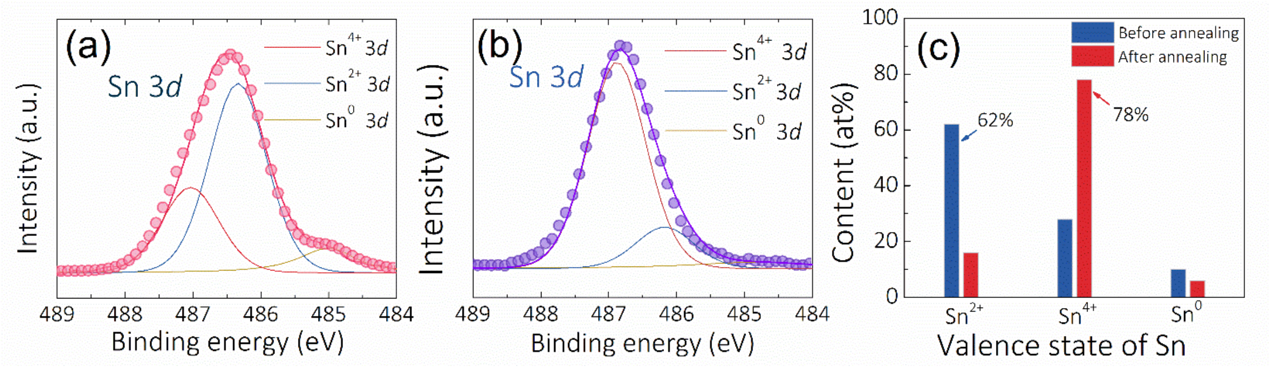

Figure S5. XPS Sn 3*d* peaks of bilayer samples. XPS Sn 3*d* peaks of (a) before and (b) after annealing bilayer samples. (c) Relative atomic contents of various valence states of tin in before and after annealing samples.

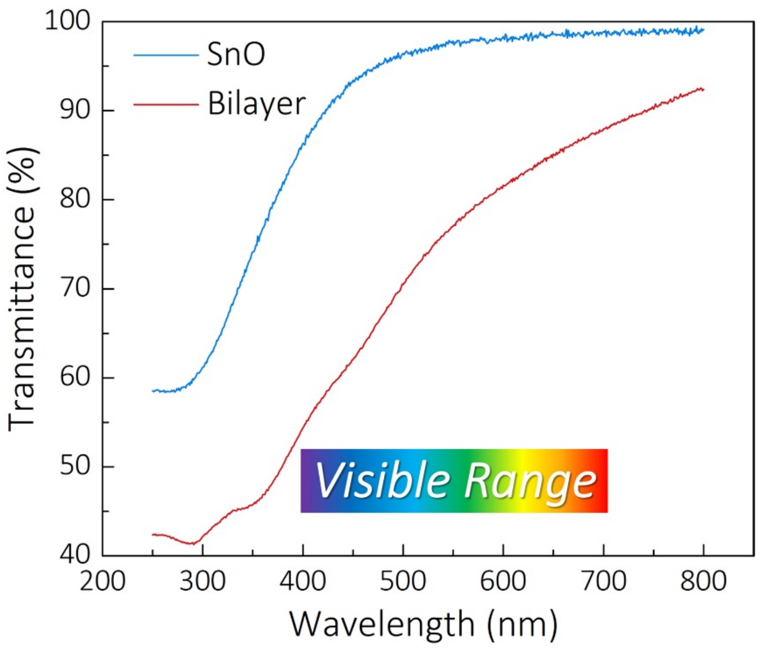

Figure S6. UV-Vis transmittance spectra. The SnO single layer film shows transmittance above 85%, and bilayer film shows transmittance above 54% in visible range.

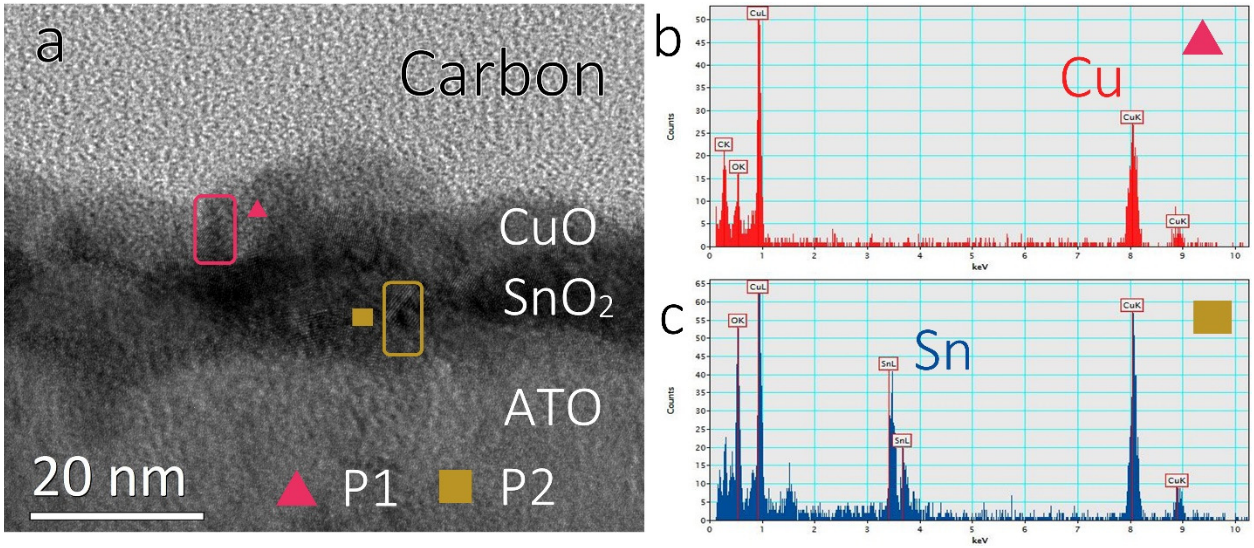

Figure S7. EDS point analysis of bilayer sample. (a) High resolution transmission electron microscopy (HRTEM) micrograph, distinct CuO (pink rectangular) and SnO_2 (yellow rectangular) zones are shown. (b) and (c) are the EDS spectra for points marked in (a), the copper signal at P2 might come from the copper grid.

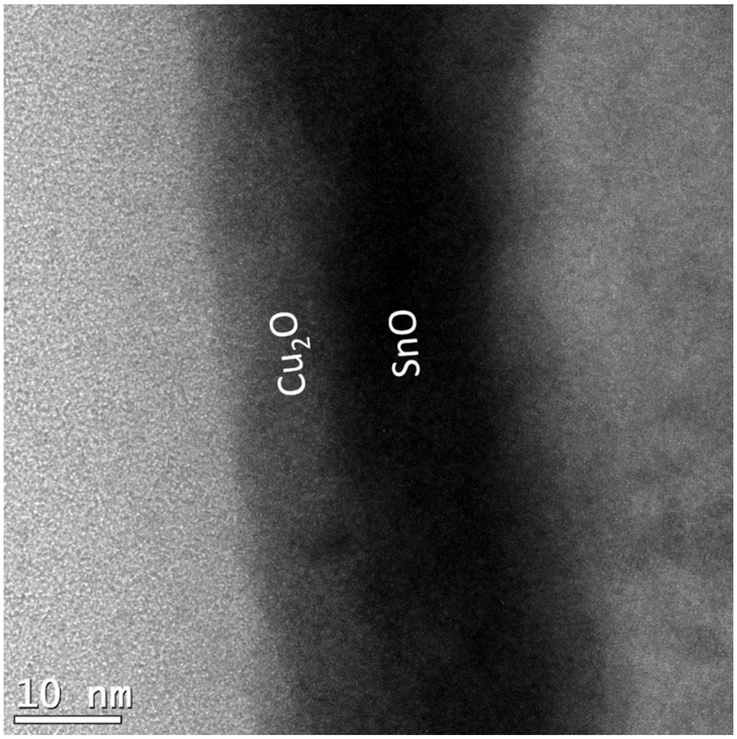

Figure S8. Cross-sectional TEM micrograph for the as-deposited bilayer sample. Both the CuO_2 and SnO layer are found to be amorphous.

Reference

[1] David R. Lide, ed., CRC Handbook of Chemistry and Physics, Internet Version 2005, <http://www.hbcpnetbase.com>, CRC Press, BocaRaton, FL (2005)
